# Phenotypic screening identifies a new oxazolone inhibitor of necroptosis and neuroinflammation

**DOI:** 10.1038/s41420-018-0067-0

**Published:** 2018-07-10

**Authors:** Sara R. Oliveira, Pedro A. Dionísio, Hugo Brito, Lídia Franco, Catarina A. B. Rodrigues, Rita C. Guedes, Carlos A. M. Afonso, Joana D. Amaral, Cecília M. P. Rodrigues

**Affiliations:** Research Institute for Medicines (iMed.ULisboa), Faculty of Pharmacy, Universidade de Lisboa, Lisboa, Portugal

**Keywords:** Drug development, Pharmacology

## Abstract

Necroptosis is a regulated form of necrosis, which may be critical in the pathogenesis of neurodegenerative diseases. Neuroinflammation, characterized by the activation of glial cells such as microglia, is closely linked with neurodegenerative pathways and constitutes a major mechanism of neural damage and disease progression. Importantly, inhibition of necroptosis results in disease improvement, unveiling an alternative approach for therapeutic intervention. In the present study, we screened a small library of new molecules, potentially inhibitors of necroptosis, using two cellular models of necroptosis. A new oxazolone, Oxa12, reduced tumour necrosis factor α (TNF-α)-induced necroptosis in mouse L929 fibrosarcoma cells. Notably, Oxa12 strongly inhibited zVAD-fmk-induced necroptosis in murine BV2 microglial cells. Moreover, Oxa12 blocked phosphorylation of mixed-lineage kinase domain-like protein (MLKL), and interfered with necrosome complex formation, indicating that Oxa12 targets components upstream of MLKL. In fact, in silico molecular docking studies revealed that Oxa12 is occupying a region similar to the 1-aminoisoquinoline type II kinase inhibitor inside the receptor-interacting protein 1 (RIP1) kinase domain. Finally, in microglial cells, Oxa12 attenuated zVAD-fmk- and lipopolysaccharide (LPS)-induced inflammatory processes, as revealed by a marked decrease of TNF-α and/or IL-1β expression. More specifically, Oxa12 negatively targeted c-Jun N-terminal kinase (JNK) and p38 mitogen-activated protein kinase (MAPK) pathways, as well as NF-κB activation. Overall, we identified a strong lead inhibitor of necroptosis that is also effective at reducing inflammation-associated events. Oxa12 is a promising candidate molecule for further development to target disease states dependent on RIP kinase activity.

## Introduction

Neurodegenerative diseases are a group of chronic disorders characterized by progressive neuronal dysfunction and loss in specific areas of the nervous system. Neuroinflammation has also emerged as a critical mechanism contributing to neuronal damage and fuelling disease progress.

Necrosis has historically been considered an accidental and passive cell death mechanism^1,2^. However, evidence now reveals that a subtype of necrosis, necroptosis, can be molecularly controlled^3^, and viewed as an appealing target for therapeutic intervention. Necroptosis is a caspase-independent form of cell death that can be activated by death receptors, particularly tumour necrosis factor receptor 1 (TNFR1), as well as Toll-like receptor 3 (TLR3), and TLR4^4,5^. Downstream signalling involves auto- and trans-phosphorylation of receptor-interacting protein 1 (RIP1) and 3 (RIP3), converging on the assembly of an amyloid-like structure, named necrosome^6^. RIP3 then recruits and phosphorylates pseudokinase mixed lineage kinase domain-like (MLKL), which in turn triggers membrane rupture, resulting in necroptotic cell death^7–9^.

The role of necroptosis in disease was first investigated in ischemic brain injury^10^. Since then, necroptosis has emerged as a critical event in the pathogenesis of other diseases, namely inflammatory diseases such as pancreatitis^11^, skin inflammation^12^, and liver injury^13^, but also neurodegenerative diseases. Indeed, necroptosis has been reported in Huntington’s disease^3^, amyotrophic lateral sclerosis^14^, multiple sclerosis^15^, Alzheimer’s disease^16^ and Parkinson’s disease^17^, while both genetic and chemical inhibition of necroptosis results in disease improvement.

Pharmacological targeting of necroptosis has been attempted using necrostatin-1 (Nec-1), a strong inhibitor of RIP1 kinase activity^10^. Other molecules targeting components of the necroptotic signalling pathway have been described^ 8,18^; however, none is available for clinical use. Here, we propose a robust in vitro model to screen for necroptosis inhibitors based on the murine BV2 microglial cell line. Oxa12 was identified as a potent inhibitor of necroptosis in BV2 cells and further confirmed in L929 cells. Moreover, Oxa12 attenuated neuroinflammation, highlighting the potential benefit of necroptosis inhibitors to halt neurodegenerative diseases.

## Results

### zVAD-fmk induces necroptosis in BV2 microglia cells

**Fig. 1:**
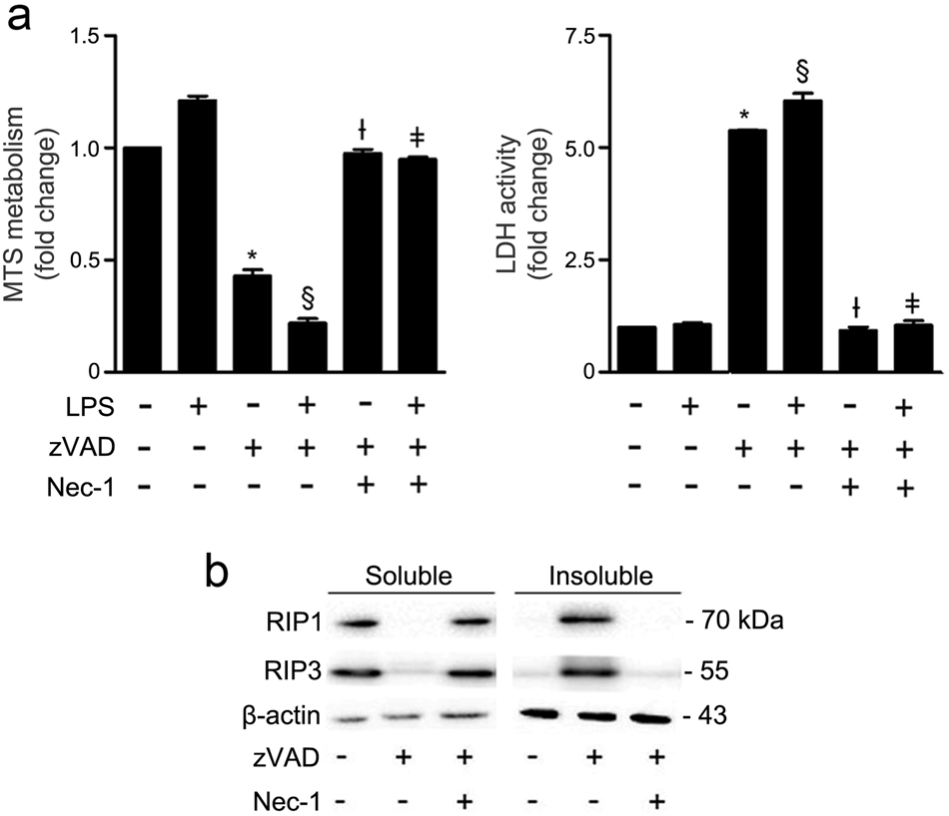
zVAD-fmk induces necroptosis in BV2 cells at 24 h of incubation. **a** BV2 cells were pre-exposed to 100 ng/mL LPS for 24 h and then incubated with the pan-caspase inhibitor zVAD-fmk (25 µM) for additional 24 h. Nec-1 (30 µM) was added 1 h before zVAD-fmk. Cell metabolic activity was determined by the MTS metabolism assay and cell membrane integrity by the LDH activity assay. Results are presented as the mean value ± SEM of three independent experiments performed in duplicates and normalized to control cells. *p < 0.001 vs. control; ^§^*p* < 0.001 vs. LPS; ^Ɨ^*p* < 0.001 vs. zVAD-fmk; ^ǂ^*p* < 0.001 vs. LPS/zVAD-fmk. **b** BV2 cells were co-incubated with zVAD-fmk (25 µM) plus Nec-1 (30 µM) for 24 h. Detergent soluble and insoluble fractions were prepared for Western blot analysis of RIP1 and RIP3. β-actin was used as loading control. Representative immunoblots are presented

Previous studies have demonstrated that primary microglia undergo RIP1/RIP3-dependent necroptosis after treatment with LPS or other TLR ligands, when caspases are inhibited^19,20^. Other authors showed that the pan- caspase inhibitor zVAD-fmk induces necroptosis in the L929 fibrosarcoma cell line by a mechanism that depends on autocrine production of TNF-α^21,22^. Here, we anticipated a new in vitro model for the study of microglial necroptosis, based on the murine BV2 microglial cell line. Exposure of BV2 cells to LPS for 48 h did not induce cell death, as detected by MTS metabolism and LDH release (Fig. 1a). However, when cells were exposed to LPS for 24 h followed by incubation with zVAD-fmk for additional 24 h, cell viability was reduced by ~80% (*p* < 0.001) with a concomitant increase in cell death. Importantly, the presence of zVAD-fmk alone was sufficient to induce high levels of cell death (*p* < 0.001). Addition of Nec-1, a RIP1-specific kinase inhibitor, fully reverted cell death to control levels in all conditions tested (*p* < 0.001), thus implicating RIP1-dependent necroptosis as the mechanism of cell death.

Necroptosis activation requires assembly of RIP1 and RIP3 in an insoluble amyloid-like complex called necrosome^6^. Exposure of cells to zVAD-fmk for 24 h, triggered RIP1 and RIP3 sequestration in the insoluble fraction (Fig. 1b), corroborating the viability data, thus confirming functional necrosome assembly and necroptosis activation. Addition of Nec-1 abolished RIP1 and RIP3 sequestration in the insoluble fraction. Taking these results into account, BV2 cells exposed to zVAD-fmk represent a robust in vitro model of microglial necroptosis, which is fully reverted when RIP1 kinase activity is inhibited by Nec-1.

### Screening for potential inhibitors of necroptosis

**Fig. 2:**
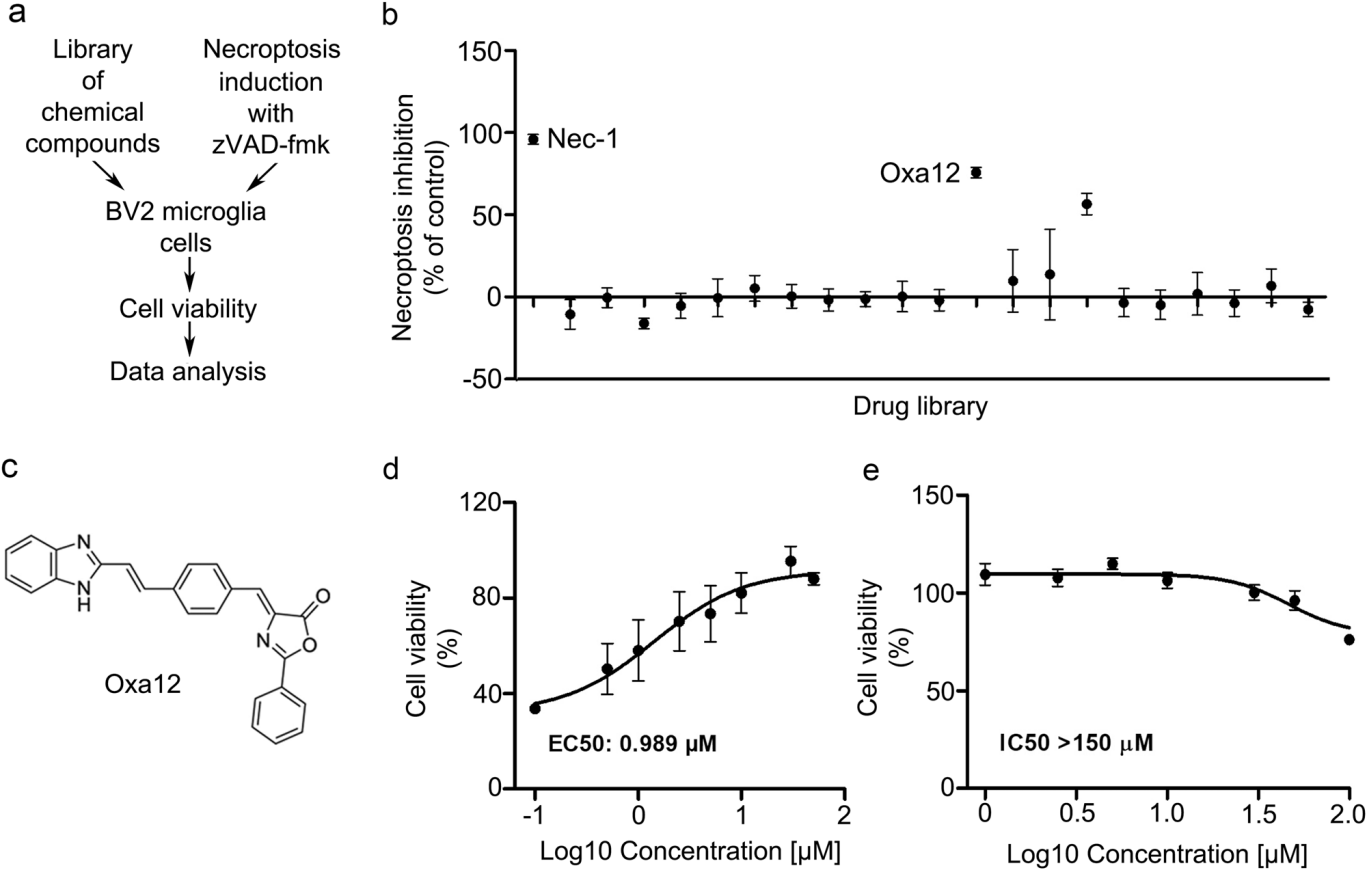
Drug screening identifies Oxa12 as necroptosis inhibitor in BV2 microglia cells. **a** Schematic overview of the drug screening workflow. **b** Normalized necroptosis inhibition values depicted as percentage of control (DMSO) for compounds (30 µM) tested on BV2 cells exposed to 25 µM zVAD-fmk for 24 h. Results are presented as the mean value ± SEM of at least three independent experiments performed in duplicates. **c** Oxa12 chemical structure. **d** BV2 cells were incubated with Oxa12 (0.1–50 µM) plus zVAD-fmk (25 µM) for 24 h. **e** BV2 cells were incubated with Oxa12 (1–150 µM) for 24 h. Cell viability was determined by the MTS metabolism assay. The results are presented as the mean value ± SEM of three independent experiments performed in duplicates and normalized to vehicle control (DMSO)

**Fig. 3:**
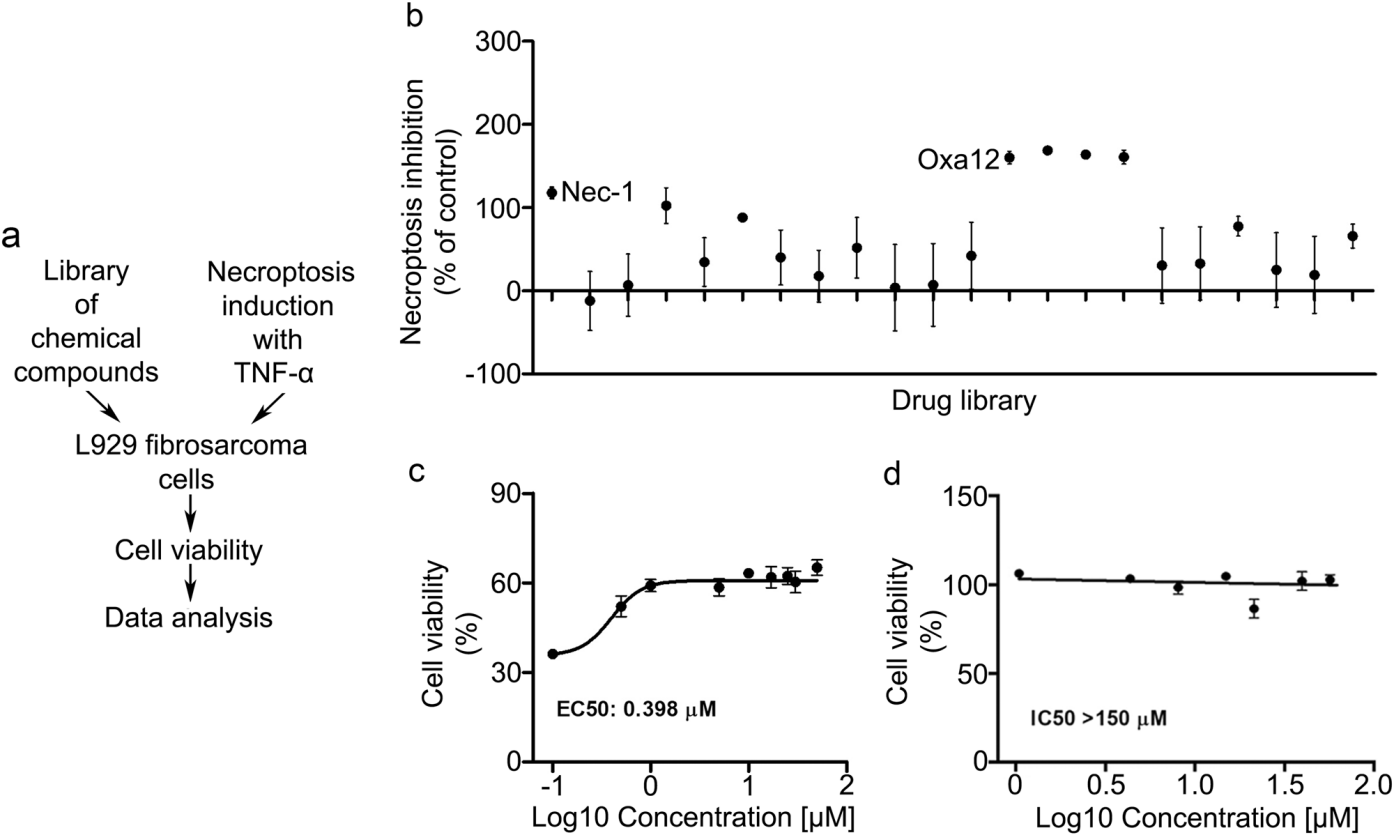
Drug screening identifies Oxa12 as necroptosis inhibitor in L929 cells. **a** Schematic overview of the drug screening workflow. **b** Normalized necroptosis inhibition values depicted as percentage of control (DMSO) for compounds (30 µM) tested on L929 cells exposed to 30 µM TNF-α for 8 h. Results are presented as the mean value ± SEM of at least three independent experiments performed in duplicates. **c** L929 cells were incubated with Oxa12 (0.1 to 50 µM) plus TNF-α (30 µM) for 24 h. **d** L929 cells were incubated with Oxa12 (1–150 µM) for 24 h. Cell viability was determined by the MTS metabolism. The results are presented as the mean value ± SEM of three independent experiments performed in duplicates and normalized to vehicle control (DMSO)

To identify novel inhibitors of necroptosis, we screened a small library of new compounds for their ability to block zVAD-fmk-induced necroptosis using the BV2 cell line model (Fig. 2a). Among the compounds tested, one potential hit was identified that significantly rescued BV2 cell viability (*p* < 0.05) (Fig. 2b). As proof-of-concept, we used a well-described model where L929 cells undergo TNF-α-induced necroptosis, which in turn is fully reverted by Nec-1 (Fig. 3a). In L929 cells, four compounds were able to revert TNF-α-induced cell death (*p* < 0.05) (Fig. 3b). We proceeded with our studies using compound Oxa12 (Fig. 2c) that showed effects similar to Nec-1 in both cell lines. Three different batches of Oxa12 were tested, showing the same ability to inhibit necroptosis in both cell models (data not shown).

To further characterize Oxa12 activity and confirm the screening data, we performed dose–response studies and quantitatively assessed its inhibitory potency. The half maximal effective concentration (EC_50_) of Oxa12 for inhibiting necroptosis was determined to be 0.989 µM in BV2 cells (Fig. 2d) and 0.398 µM in L929 cells (Fig. 3c). Drug toxicity was also assessed by determining the half maximal inhibitory concentration (IC_50_) in BV2 and in L929 cells. Notably, Oxa12 displayed no cytotoxicity throughout the whole range of concentrations in both cell lines (Figs. 2e and 3d), with IC_50_ values greater than 150 µM, highlighting the wide window of opportunity for inhibiting necroptosis with this compound.

**Fig. 4:**
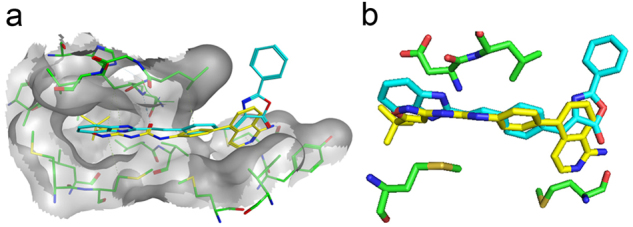
In silico molecular docking calculations for Oxa12. **a** Optimal poses obtained inside RIP1 active site (grey) for compound Oxa12 (represented in stick model and coloured blue) compared with crystallographic ligand 1-aminoisoquinoline inhibitor (PDBID: 4NEU) (yellow). **b** Compound Oxa12 and 4NEU co-crystallized inhibitor interacting with Asp156, Leu157, Met67, and Met95. Docking calculations were performed using the X-ray structure obtained for RIP1 complexed with 1-aminoisoquinoline inhibitor at resolution of 2.57 Å, PDBID: 4NEU, by the GOLD 5.2 software

Motivated by these results and to get further insight into the mechanism of action of Oxa12 at the molecular level, we performed in silico molecular docking calculations for Oxa12 inside the RIP1 kinase domain using the 4NEU X-ray structure obtained for this enzyme complexed with 1-aminoisoquinoline type II kinase inhibitor^23^. Our results revealed that without any constrain, Oxa12 is occupying a region similar to the co-crystallized inhibitor, with the phenyl rings from both compounds almost overlapped, suggesting a similar interaction pattern (Fig. 4a). Oxa12, however, is slightly rotated in the binding pocket when compared with the crystallographic ligand, being close to Asp156, Leu157, Met67, and Met95, which may enable important hydrogen bonds and π interactions. Oxa12 showed slightly increased interaction distances in comparison to the crystallographic inhibitor (Fig. 4b).

### Oxa12 is a potent inhibitor of necroptosis

**Fig. 5:**
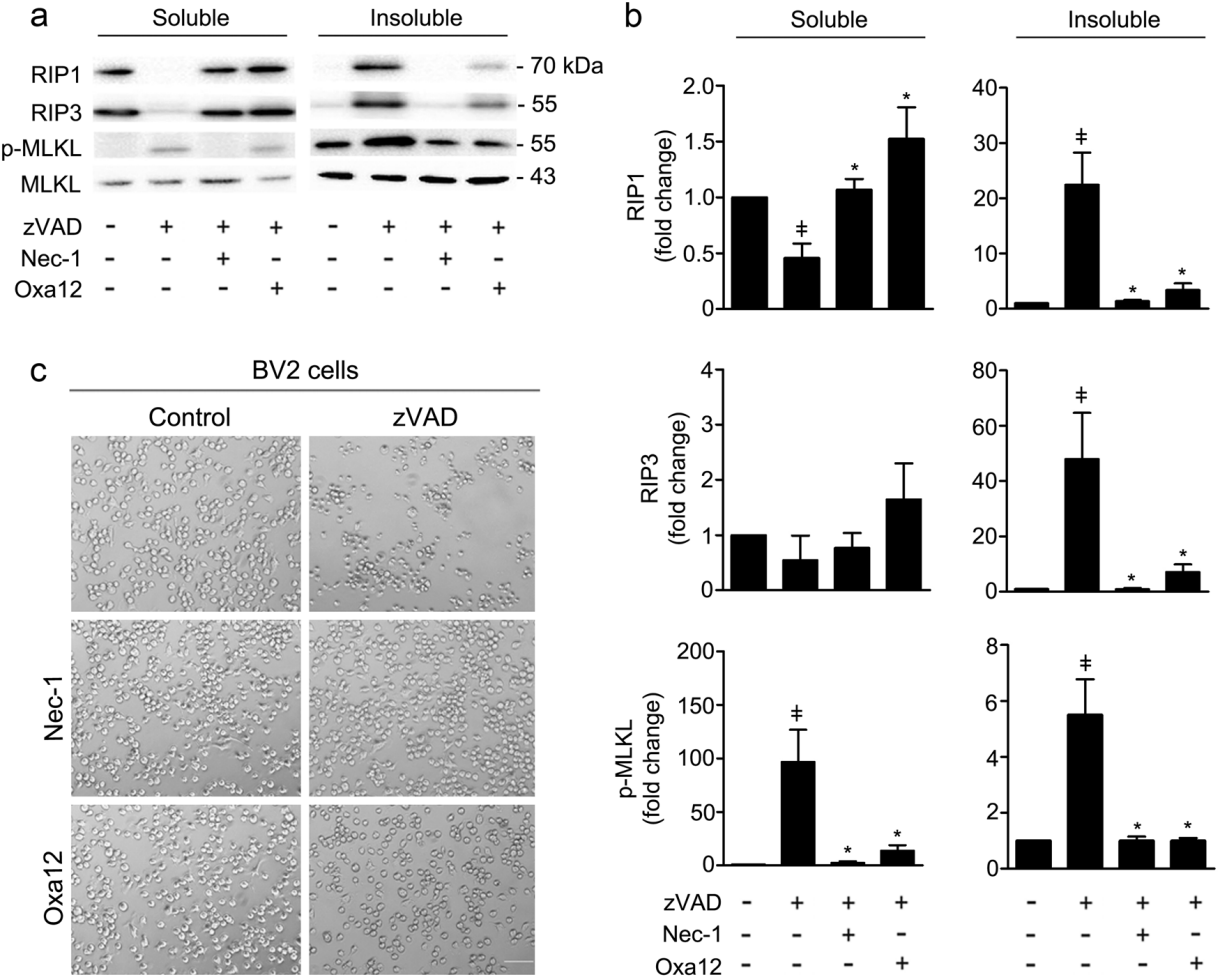
Oxa12 inhibits necroptosis in a murine microglial cell line. **a** BV2 cells were incubated with zVAD-fmk (25 µM), zVAD-fmk (25 µM) plus Nec-1 (30 µM), or zVAD-fmk (25 µM) plus Oxa12 (30 µM) for 24 h. Detergent soluble and insoluble protein fractions were prepared for Western blot analysis of RIP1, RIP3, MLKL, and p-MLKL. Representative immunoblots are presented. β-actin was used as loading control. **b** Densitometric analysis. Values are expressed as mean ± SEM of three independent experiments. ^ǂ^*p* < 0.05 vs. control; *p < 0.05 vs. zVAD-fmk. **c** Bright-field microscopic images of BV2 cells incubated with zVAD-fmk (25 µM) in the presence of absence of Nec-1 (30 µM) or Oxa12 (30 µM) for 24 h. Microscopy photographs were taken at ×100 with a Primo Vert microscope. Scale bar=100 µm

To determine the effect of Oxa12 in the necroptotic signalling pathway, we co-incubated BV2 cells with zVAD-fmk and Oxa12 for 24 h and evaluated necrosome assembly and MLKL phosphorylation in the detergent-insoluble proteome. During necroptosis, MLKL is phosphorylated at Thr357/Ser358 residues (p-MLKL) by RIP3 kinase. Once phosphorylated, MLKL oligomerizes and ultimately induces cell membrane disruption, being an excellent marker of necroptosis commitment^15^. Exposure of BV2 cells to zVAD-fmk promoted the sequestration of all key components of the necroptosis machinery, RIP1, RIP3, and p-MLKL, in the insoluble fraction (*p* < 0.01) (Fig. 5a, b). Nec-1 abrogated both necrosome assembly and MLKL phosphorylation (*p* < 0.01). Importantly, Oxa12 also abolished all necroptosis-associated changes (*p* < 0.05).

Microscopy analysis of cell morphology was consistent with the previous results, showing evident cell death after zVAD-fmk treatment and improvement of this phenotype when Nec-1 or Oxa12 were added to BV2 (Fig. 5c). Of note, neither Nec-1 nor Oxa12 alone affected cell morphology. The effect of Oxa12 was further confirmed in L929 cells, in which Oxa12 prevented TNF-α-induced increase of p-MLKL (*p* < 0.05) and preserved cell morphology (Suppl. Figure 1a and b). Taken together, these results implicate Oxa12 as a strong inhibitor of necroptosis.

### Oxa12 reduces TNF-α gene expression and secretion

**Fig. 6:**
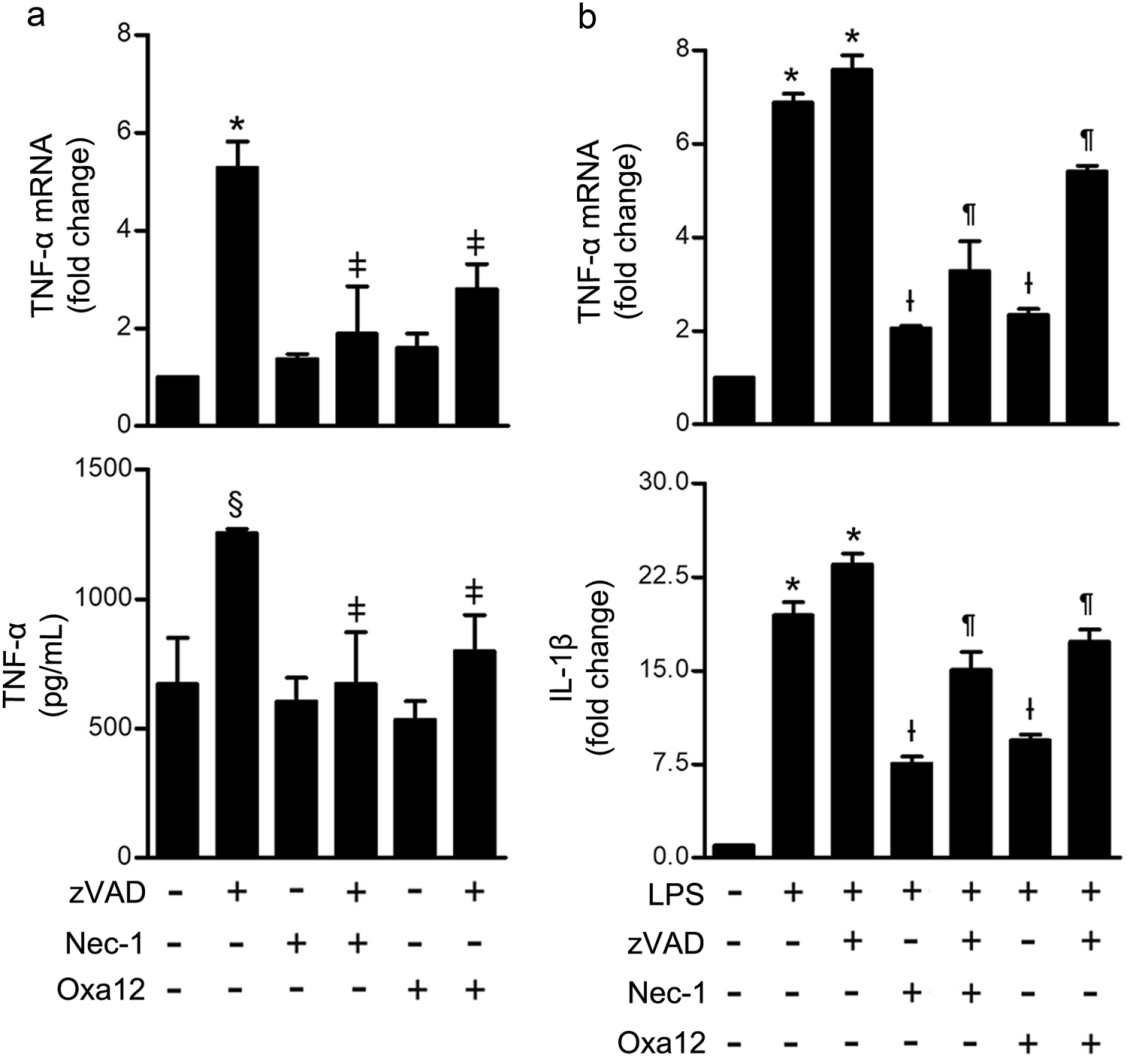
Oxa12 decreases TNF-α gene expression and protein secretion levels. **a** BV2 cells were incubated with zVAD-fmk (25 µM) in the presence or absence of Nec-1 (30 µM) or Oxa12 (30 µM) for 24 h. TNF-α mRNA levels were measured by qRT-PCR and secreted TNF-α by ELISA. Results are expressed as mean ± SEM from three independent experiments. *p < 0.001 vs. control; ^ǂ^*p* < 0.001 vs. zVAD-fmk. **b** BV2 cells were incubated with 100 ng/mL LPS, LPS plus zVAD-fmk (25 µM) in the presence or absence of Nec-1 (30 µM) or Oxa12 (30 µM) for 24 h. TNF-α and IL-1β mRNA levels were measured and results are expressed as mean ± SEM from three independent experiments. *p < 0.001 vs. control; ^Ɨ^*p* < 0.001 vs. LPS; ^¶^*p* < 0.001 vs. LPS/zVAD-fmk

Necroptosis is a proinflammatory type of cell death that culminates in the release of intracellular components to the extracellular space^24^. Moreover, zVAD-fmk-induced necroptosis in L929 cells is dependent on the production and autocrine secretion of TNF-α^22^. To determine whether Oxa12-mediated inhibition of necroptosis may also contribute to decrease necroptosis-associated inflammation, we analysed mRNA levels of proinflammatory genes, including cyclooxygenase 2 (*COX2*), interleukin-6 (*IL-6*), nucleotide-binding oligomerization domain-like receptor (NLR) pyrin domain containing 3 (*NLRP3*), and *TNF-α*, as well as TNF-α protein secretion to the culture medium. *COX2* and *NLRP3* did not show any significant variation, with either zVAD-fmk alone or in combination with Nec-1/Oxa12, while *IL-6* was barely detectable in all conditions tested (data not shown). In contrast, exposure of BV2 cells to zVAD-fmk for 24 h resulted in a significant increase of TNF-α gene expression (*p* < 0.001) and protein secretion levels (*p* < 0.05) (Fig. 6a). Importantly, at the mRNA level, this increase was significantly reduced upon Nec-1 and Oxa12 incubation (*p* < 0.05). Similarly, Nec-1 completely abolished zVAD-fmk-induced TNF-α secretion, while Oxa12 diminished TNF-α levels in the culture medium by about 70% (*p* < 0.05). These results suggest that, similarly to what was described for L929 cells, the autocrine secretion of TNF-α is a key step in zVAD-fmk-induced necroptosis in BV2 microglia cells, and Oxa12 appears to counteract this proinflammatory condition.

To determine if Oxa12 could play a role in protecting BV2 microglia cells from an inflammatory stimulus, per se, we investigated *TNF-α* and *IL-1β* gene expression after stimulation of BV2 cells with LPS. As expected, exposure of BV2 cells to LPS significantly increased *TNF-α* and *IL-1β* gene expression (*p* < 0.001), an effect exacerbated by LPS/zVAD-fmk co-incubation (*p* < 0.001) (Fig. 6b). Moreover, Nec-1 robustly reduced *TNF-α* and *IL-1β* mRNA levels (*p* < 0.001), which is in accordance with previous studies^25–27^. Notably, Oxa12 partially reverted LPS- and LPS/zVAD-fmk-induced *TNF-α* and *IL-1β* gene expression (*p* < 0.001), thus highlighting the anti-inflammatory potential of this compound.

### Oxa12 inhibits zVAD-fmk-induced JNK, p38 MAPK and NF-κB activation

**Fig. 7:**
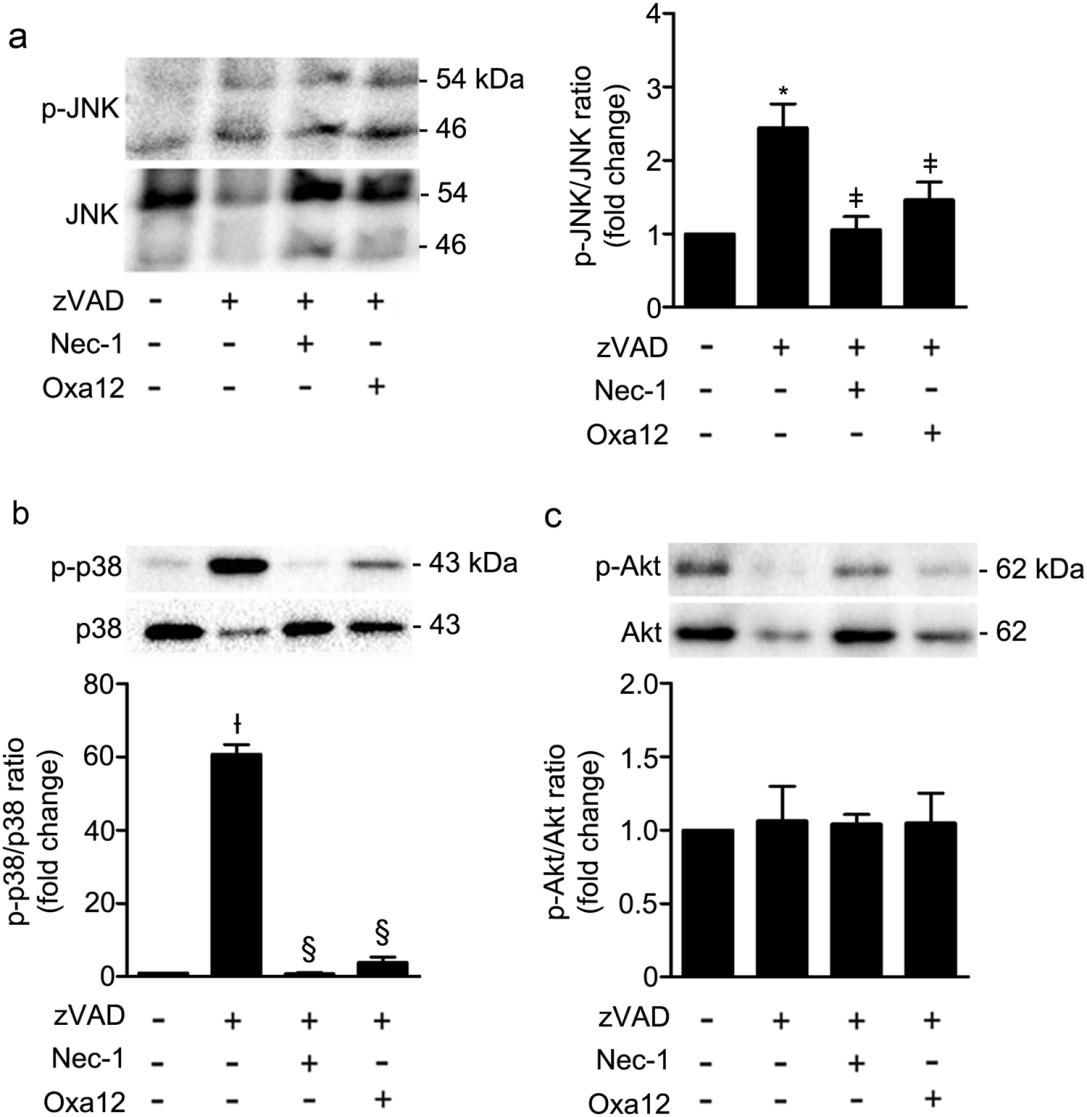
Oxa12 decreases zVAD-fmk-induced JNK and p38 MAPK activation in BV2 cells. BV2 cells were treated with zVAD-fmk (25 µM) in the presence or absence of Nec-1 (30 µM) or Oxa12 (30 µM) for 24 h. Representative immunoblots are presented, together with the respective densitometric analysis of the p-JNK/JNK, p-p38/p38 and p-Akt/Akt ratios. **a** p-JNK (Thr183/Tyr185) and total JNK. **b** p-p38 (Thr180/Tyr182) and total p38. **c** p-Akt (Ser473) and total Akt. Results are expressed as mean ± SEM of three independent experiments. *p < 0.001 vs. control; ^ǂ^*p* < 0.05 vs. zVAD-fmk; ^Ɨ^*p* < 0.01 vs. control; ^§^*p* < 0.001 vs. zVAD-fmk

To further dissect which inflammatory pathways Oxa12 specifically targets, we evaluated JNK (Thr183/Tyr185) and p38 (Thr180/Tyr182) phosphorylation, two classic MAPK inflammatory signalling pathways. We also evaluated phosphorylation of protein kinase B, also known as Akt (Ser473). Indeed, others have shown that JNK and Akt, when activated, have important roles in necroptosis, being involved in the production and autocrine secretion of TNF-α^21^. Incubation of BV2 cells with zVAD-fmk for 24 h induced a significant increase in JNK and p38 phosphorylation, thus suggesting activation of these two signalling pathways (Fig. 7a, b). In contrast, Nec-1 completely abolished JNK and p38 phosphorylation, while treatment with Oxa12 markedly reduced their activation. No significant changes were observed for Akt activation under these experimental conditions (Fig. 7c).

**Fig. 8:**
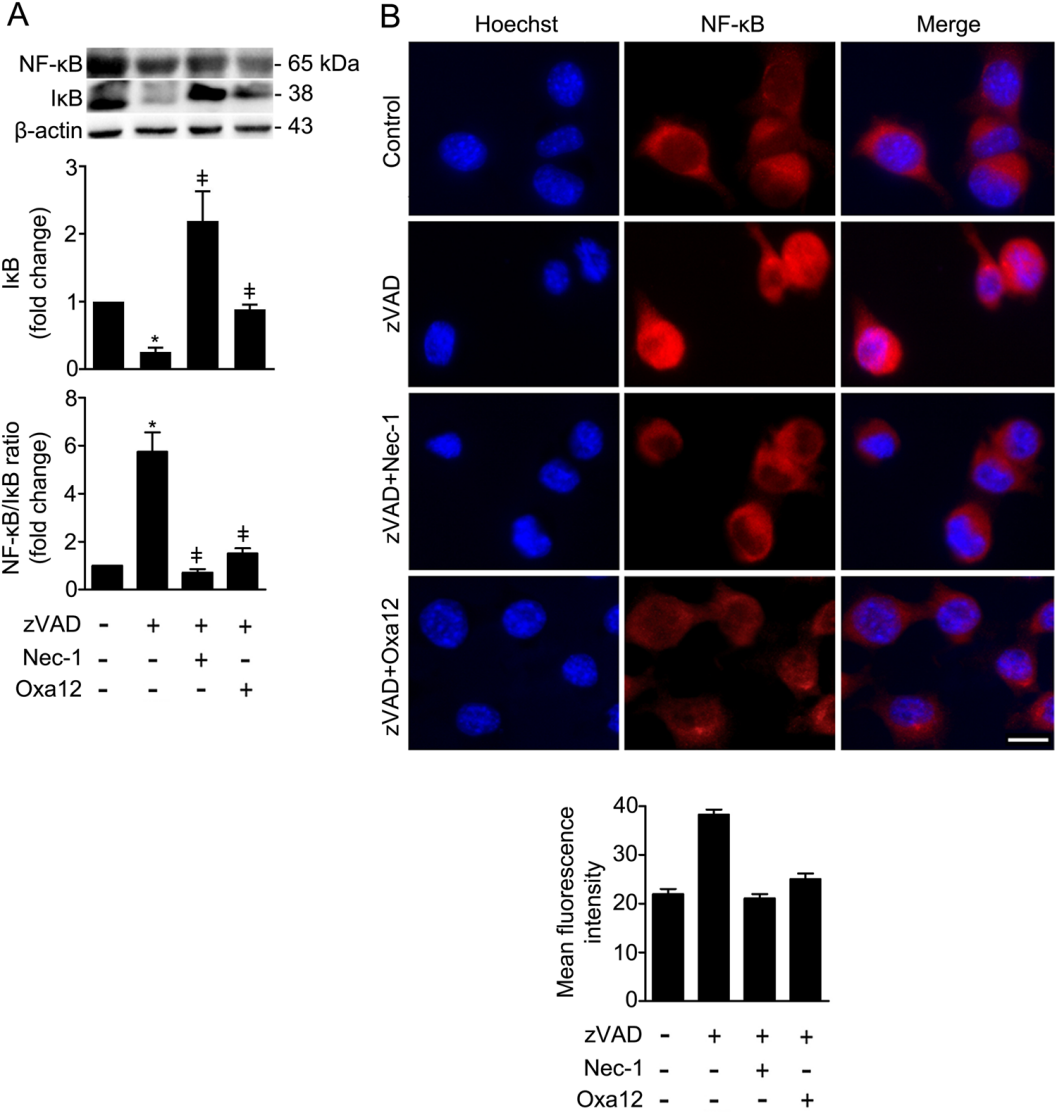
Oxa12 reduces NF-κB/IκB ratio and NF-κB p65 nuclear translocation when compared to zVAD-fmk-treated cells. **a** BV2 cells were incubated with zVAD-fmk (25 μM) in the presence or absence of Nec-1 (30 µM) or Oxa12 (30 µM) for 24 h. Representative immunoblots of total NF-κB are presented together with the respective densitometric analysis of NF- κB/IκB ratio. β-actin was used as loading control. Results are expressed as mean ± SEM from three independent experiments. *p < 0.05 vs. control; ^ǂ^*p* < 0.05 vs. zVAD-fmk. **b** Representative images of immunofluorescence staining showing NF-κB p65 (red) nuclear translocation in BV2 cells treated with zVAD-fmk (25 µM) in the presence or absence of Nec-1 (30 µM) or Oxa12 (30 µM) for 5 h, and quantification of mean fluorescence intensity. Cell nuclei were detected by Hoechst (blue). Scale bar=1 µm

NF-κB has long been considered a pivotal mediator of inflammatory responses^27^, largely based on its activation by proinflammatory cytokines such as TNF-α. We investigated whether NF-κB signalling was activated in BV2 cells after exposure to zVAD-fmk. No significant differences were observed in NF-κB steady-state levels upon exposure of BV2 cells to zVAD-fmk (Fig. 8a). However, IκB was markedly decreased (*p* < 0.01) with a concomitant increase of the NF-κB/IκB ratio (*p* < 0.01), suggesting that zVAD-fmk treatment induced NF-κB activation (Fig. 8a). Importantly, NF-κB activation was further confirmed by immunofluorescence. BV2 cells treated with zVAD-fmk for 5 h showed a marked increase of NF-κB p65 subunit in the nucleus (Fig. 8b). In contrast, both Nec-1 and Oxa12 strongly suppressed zVAD-fmk-driven NF-κB activation (*p* < 0.01).

## Discussion

Neurological disorders ranked as the leading cause group of disability, and the second-leading cause of mortality worldwide^28^. Globally, the burden of neurodegenerative diseases is expected to increase over the next years, mostly because of the expanding population and ageing. Currently, the treatments available are ineffective. Therefore, interventions that slow or stop neurodegeneration are an urgent, unmet need. Although apoptosis is frequently implicated in neurodegeneration, necroptosis has been recently described as a prominent player in neurodegenerative disease pathobiology^3^. In fact, the role of necroptosis has been studied in the pathogenesis of a broad spectrum of diseases, including neurodegenerative diseases, where inhibition of necroptosis is considered a beneficial event^14–17^. Neuroinflammation is also a pathological hallmark of neurodegenerative diseases, where microglia have a fundamental role in regulating innate and adaptive immune responses^29,30^. Recently it was shown that in certain pathological conditions, such as multiple sclerosis, microglia present defective caspase-8 activation, which may promote inflammation through activation of necroptosis, thus contributing to disease progression^15^. Others have also shown that necroptosis in retina microglia triggers neuroinflammation and exacerbates retinal neural damage and degeneration in mice^25^. In these cases, targeting the necroptotic machinery has been proven to be useful to attenuate microglia-mediated neuroinflammation and ameliorate neural injury.

Here, we used the murine BV2 microglia cell line as a new and robust cellular model to screen for potential small molecule modulators of microglial necroptosis. Our results showed that BV2 cells undergo necroptosis after 24 h incubation with the pan- caspase inhibitor, zVAD-fmk, which is in agreement with previous studies reporting that zVAD-fmk induces necroptosis in L929 cells^21,22^. Nec-1, a RIP1 kinase inhibitor here used as positive control of necroptosis inhibition, fully reverted cell death to control levels, thus implicating RIP1-dependent necroptosis as the death mechanism. Interestingly, pre-incubation with LPS, a well-known TLR4 agonist, accelerated the death mechanism, thus linking necroptosis to this inflammatory context. Indeed, we observed a further decrease of ~30% in MTS metabolism when cells were pre-incubated with LPS for 24 h and, then, incubated with zVAD-fmk for additional 24 h, while no differences were observed in LDH activity. These results suggest that cell membrane permeabilization and leakage of intracellular components, such as LDH, may be an initial step in the necroptotic cascade, while mitochondria dysfunction appears to contribute indirectly to late-stage necroptosis^9,31^. In addition, we observed that zVAD-fmk alone induced necrosome assembly after 24 h. It is known that zVAD-fmk triggers the production of TNF-α at the transcriptional level, and subsequently the autocrine secretion of this cytokine, which in turn may activate TNFR to induce necroptosis^22^. Reversion of necrosome formation induced by Nec-1 further confirmed the importance of this molecular platform as the cell death inducer in this model. Overall, BV2 cells exposed to zVAD-fmk represent a robust in vitro model of microglial necroptosis, along with full reversion of all necroptotic processes when RIP1 kinase activity is inhibited by Nec-1.

Since the discovery of Nec-1 as the first inhibitor of necroptosis^10^, other inhibitors have been described, including RIP1, RIP3 and MLKL inhibitors. Nonetheless, all compounds identified so far show several limitations. Nec-1 itself is highly effective in inhibiting necroptosis; however, it has inadequate pharmacokinetic properties including very short in vivo half-life of ~1 h, reduced solubility^32^, and presents off-target activity against indoleamine-pyrrole 2,3-dioxygenase (IDO), a modulator of the innate and adaptive immune system^33,34^, serine/threonine-protein kinase 1 (PAK1) and cAMP-dependent protein kinase catalytic subunit α (PKAcα). Later, Nec-1 optimizations led to the identification of Nec-1s (Nec-1 stable), also known as 7-Cl-O-Nec-1, which is a selective RIP1 kinase inhibitor with low toxicity. However, this molecule has also poor pharmacokinetic properties^33,34^. Regarding RIP3 inhibitors, all compounds described so far also induce apoptosis^18^. Necrosulfonamide (NSA), a well-known MLKL inhibitor, inhibits necroptosis by blocking human MLKL phosphorylation, but not the rodent homolog, thus invalidating pharmacological, pharmacokinetic and toxicity preclinical testing^8^. In sum, although tool compounds blocking necroptosis have previously been developed, no necroptosis inhibitors are in clinical use to date. Therefore, the discovery of new specific and potent pharmacologic inhibitors of necroptosis is relevant and of utmost importance.

In this study, we screened a library of new compounds that potentially modulate necroptosis. We identified one hit, Oxa12, that inhibits necroptotic cell death in BV2 cells (EC_50_ = 0.989 µM) and L929 cells (EC_50_ = 0.459 µM) without cytotoxicity associated. Further, Oxa12 inhibited necroptosis-associated events in murine BV2 cells, including necrosome assembly and MLKL S358 phosphorylation, two key markers of necroptosis commitment. Importantly, the docking pose of Oxa12 inside RIP1 kinase active site is similar to that of the co-crystallized 1-aminoisoquinoline RIP1-specific inhibitor, thus highlighting the potential of Oxa12 as a RIP1 inhibitor.

To further characterize the mechanism of action of Oxa12 and because necroptosis is an inflammatory type of cell death, we hypothesized that necroptosis inhibition by Oxa12 could also result in decreased inflammation. Our results showed that BV2 cells exposed to zVAD-fmk presented increased levels of *TNF-α* gene expression and cytokine secretion. These findings were in accordance with previous studies reporting the production and autocrine secretion of TNF-α as a crucial factor in zVAD-fmk-induced necroptosis^22,35^. No differences were detected in *COX2* and *NLRP3* transcription levels, which might be explained by the involvement of these two proteins in later stages of the inflammatory cascade, being extensively regulated by other proteins and signalling pathways^36,37^. Similarly to what happens with Nec-1, treatment of BV2 cells with Oxa12 reduced TNF-α gene expression and cytokine secretion levels, suggesting that RIP1 kinase activity is required for TNF-α production, as previously reported^21^.

After confirming the involvement of necroptosis-associated inflammation in our cellular model and its concomitant reduction by Oxa12, we further investigated if Oxa12 could protect BV2 microglial cells from a classic inflammatory stimulus, independent of necroptosis activation. Therefore, we used LPS, a specific ligand of TLR4 that activates downstream pro-inflammatory signalling cascades without eliciting cell death. As expected, treatment of BV2 cells with LPS for 24 h induced *TNF-α* and *IL-1β* gene expression. Curiously, Nec-1 counteracted the effect of LPS implicating RIP1 in the production of proinflammatory cytokines, unrelated to necroptosis. In fact, this was not the first time that an anti-inflammatory role was reported for Nec-1, independently of its activity as necroptosis inhibitor^38^. Similarly, Oxa12 strongly inhibited LPS-induced *TNF-α* and *IL-1β* mRNA levels, thus revealing the potential of this molecule not only as an inhibitor of necroptosis, but also as an agent with ability to resolve established inflammation. Exposure of LPS-stimulated BV2 cells to zVAD-fmk further potentiated the transcription of proinflammatory *TNF-α* and *IL-1β*, suggesting that caspase blockade sensitizes cells to LPS challenge. Nonetheless, both Nec-1 and Oxa12 were still capable of reducing LPS/zVAD-fmk-induced pro-inflammatory mediators.

To better understand which inflammatory pathways Oxa12 specifically targeted, we evaluated the activation of two MAPK signalling pathways, JNK and p38, as well as the activation of Akt. Importantly, activated MAPK signalling pathways are described in the pathogenesis of neurodegenerative diseases, including Alzheimer’s disease and Parkinson’s disease as contributors of inflammation and neuronal death^39–41^. Deregulation of Akt pathway is also reported in brains from Alzheimer’s and Parkinson’s disease patients^42^. Recent studies have reported that JNK activation plays an important role during zVAD-fmk-induced necroptosis in L929 cells downstream to RIP1 kinase, promoting TNF-α gene expression^21,22^. Increased TNF-α transcription may translate into elevated levels of this cytokine, which could induce necroptosis through TNFR activation^22^. Our results are in line with these studies, since exposure of BV2 cells to zVAD-fmk induced high levels of JNK activation, which may be related with the increase observed in TNF-α gene expression and cytokine secretion levels. By contrast, Nec-1 and Oxa12 abolished both JNK activation and TNF-α gene expression and cytokine secretion levels, thus confirming the involvement of this signalling pathway in zVAD-fmk-induced necroptosis. Regarding p38, while some authors reported that this signalling pathway is not activated during necroptosis in L929 cells^43^, others show that pharmacological inhibition of TNF-α-induced necroptosis in L929 cells resulted in p38 activation^44^. Here, we show that treatment of BV2 cells with zVAD-fmk induced a marked increase in p38 phosphorylation, which is fully reverted by Nec-1 and Oxa12, suggesting activation of this signaling pathway during zVAD-fmk-mediated necroptosis in BV2 microglial cells. It is well known that JNK and p38 MAPK pathways are activated by several inflammatory mediators in different cell lines, being involved in stress responses and inflammation^45^, being possible that the intracellular components released by necroptotic cells may induce JNK and p38 MAPK activation.

The role of Akt in necroptosis has also been demonstrated. Akt is linked to necroptosis in L929 cells, where it plays a key role in mediating TNF-α synthesis^46^. Inhibition of Akt protected L929 cells from TNF-α-induced necroptosis and triggers autophagy instead^46,47^. In contrast, others showed that zVAD-fmk alone is capable of inducing Akt activation, with this activation being dependent of Thr308 phosphorylation, with no alterations observed in Akt Ser473 phosphorylation^48^. In the present study, we evaluated Akt Ser473 phosphorylation as a marker of Akt activation, which may explain the absence of significant differences between the conditions tested.

In the nervous system, evidence supports a dual role of NF-κB in neurodegenerative diseases. In general, it appears that activation of NF-κB in neurons protects against degeneration, whereas activation in glial cells mediates pathological inflammatory processes^49^. Taking this into account, we were not surprised by the activation of NF-κB in BV2 cells treated with zVAD-fmk. In contrast, Nec-1 and Oxa12 strongly abrogated this activation, which highlights their potential at reducing inflammation-associated events. Altogether, our findings suggest that zVAD-fmk induces the expression of inflammatory target genes at early time-points, thus promoting the downstream activation of important inflammatory signalling pathways. Further, longer incubation times will activate necroptosis, which has also a prominent role on inflammation^50^.

Overall, we established a robust in vitro model of microglia necroptosis, based on the murine BV2 microglial cell line and identified a strong lead inhibitor of this type of regulated cell death. Oxa12 is efficient at decreasing necroptosis-driven inflammation, as well as activation of important signalling pathways including JNK, p38 MAPK and NF-κB. Thus, Oxa12 can now be considered a promising candidate molecule for targeting pathologies-dependent on RIP1-kinase activity.

## Materials and methods

### Cell culture and reagents

BV2 murine microglia cells (kindly provided by Elsa Rodrigues, University of Lisbon) were cultured in RPMI 1640 medium (GIBCO® Life Technologies, Inc. Grand Island, USA), supplemented with 10% heat inactivated fetal bovine serum (FBS), 1% antibiotic/antimycotic solution and 1% GlutaMAX^TM^ (GIBCO). Throughout experiments, the culture media was replaced by RPMI supplemented with 1% antibiotic/antimycotic solution, 1% insulin-transferrin-selenium (RPMI/ITS) and 1 mg/mL bovine serum albumin (BSA; GIBCO). The L929 murine fibrosarcoma cell line (kindly provided by Junying Yuan, Harvard Medical School) was cultured in DMEM (GIBCO) supplemented with 10% FBS and 1% GlutaMAX^TM^. Cells were maintained at 37 °C in a humidified atmosphere of 5% CO_2_. Other chemicals used were as follows: LPS from *Escherichia coli* 055:B5 (#437625; Calbiochem, San Diego, CA, USA), Nec-1 (Sigma-Aldrich, St. Louis, MO, USA), dimethyl sulfoxide (DMSO; Sigma-Aldrich), Z-Val-Ala-Asp-fluoromethylketone (zVAD-fmk) pan- caspase inhibitor (Enzo Life Sciences, Farmingdale, NY, USA), and recombinant murine TNF-α (PeproTech EC Ltd., London, UK). A small in-house library of potential inhibitors of necroptosis was tested, including Oxa 12 ((Z)-4-(4-((E)-2-(1H-benzo[d]imidazol-2-yl)vinyl) benzylidene)-2-phenyloxazol-5(4H)-one) that was synthetized accordingly to a reported method^51,52^.

### Viability assay

BV2 cells were plated in 96-well plates at 5 × 10^3^ cells/well. After 24 h of cell plating, media was replaced by fresh RPMI/ITS containing 100 ng/mL LPS, or no addition, and cells were incubated for additional 24 h. Then, BV2 cells were exposed to 25 µM zVAD-fmk for additional 24 h. Nec-1 (30 µM) was added 1 h before zVAD-fmk. Cellular metabolic activity was measured using the CellTiter 96® Aqueous Non-Radioactive Cell Proliferation (MTS) Assay (Promega, Madison, WI, USA). Changes in absorbance were measured at 490 nm using GloMax® Multi Detection System (Sunnyvale, CA, USA).

### General cell death assays

Cell membrane integrity was evaluated using the lactate dehydrogenase (LDH) Cytotoxicity Kit^PLUS^ (Roche Diagnostics GmbH, Mannheim, Germany). Briefly, 50 μL of cell supernatants were incubated with 50 μL of assay substrate for 10–30 min, at room temperature, protected from light. Absorbance readings were measured at 490 nm, with 620 nm reference wavelengths using a Bio-Rad Model 680 microplate reader. Further, cell death was also determined using the ToxiLight^TM^ BioAssay Kit (Lonza Walkersville Inc., Walkersville, MD, USA), according to the manufacturer’s instructions. The release of adenylate kinase enzyme from damaged cells was determined using 10 µL each of cell supernatants and bioluminescent cytolysis assay in the microplate reader.

### Drug screening

Drug screening was performed using BV2 and L929 cell lines. BV2 cells were seeded in 96-well plates at 7 × 10^3^ cells/well; necroptosis was induced using 25 µM zVAD-fmk and compounds were incubated at a final concentration of 30 µM. Cell viability and death were assessed 24 h later by MTS and LDH assays. For the L929 cell line, cells were seeded in 384-well plates at 1 × 10^3^ cells/well, necroptosis was induced using 30 µM TNF-α, and compounds were incubated at a final concentration of 30 µM. Cell death was assessed 8 h later using the ToxiLight™ BioAssay Kit. All measurements were performed in duplicate. A percentage of control was calculated to normalise for variability across different plates. Half-maximum effective concentration (EC_50_) at inhibiting necroptosis and half maximal inhibitory concentration (IC_50_) were calculated for the selected hits in both cell lines, using the GraphPad Prism Software version 5.00 (GraphPad Software, Inc., San Diego, CA, USA) with the log (inhibitor) vs. response (variable slope) function.

### Docking studies

Non-covalent molecular docking calculations were used to understand compound activity against RIP1. 3D structure coordinates of RIP1 were obtained from five different crystal structures at the Protein Data Bank (PDB), 4ITI, 4ITH, 4ITJ, and 4NEU, with a resolution in the range 1.80–2.89 Å. Co-crystallized inhibitors and all crystallographic waters were removed. Hydrogen atoms were then added and the protonation states were correctly assigned using the Protonate-3D tool within the Molecular Operating Environment (MOE) 2016.08 software package. All the compounds tested were built and energy minimized using Amber forcefield implemented in MOE 2016.08 software.

Molecular docking studies were then performed using the GoldScore scoring function from GOLD 5.2 software package and each ligand was subjected to 1000 docking runs. For all five structures, the docking protocol was validated using crystallographic ligands; poses were reproducible with RMSD´s lower than 1 Å. In addition, an extra validation of the receptor structure was performed with 10 established RIP1 inhibitors, including Nec-1 and derivatives, GSK962, GSK963, and ponatinib. Significant differences were observed between final poses and scores obtained using 4ITI, 4ITH, 4ITJ, and 4NEU. The 4NEU structure was able to reproduce the experimental activities, while all other structures showed severe penalties in scores due to a very tight active centre. Results presented here were obtained with the 4NEU prepared structure.

### Total and soluble/insoluble protein extraction

For total protein extraction, BV2 cells were plated in 60 mm culture dishes at 4 × 10^5^ cells/dish, and L929 cells were plated in 6-well plates at 2.5 × 10^5^ cells/well. Floating and adherent cells were collected directly in nonyl phenoxypolyethoxylethanol (NP-40) lysis buffer (1% NP-40, 20 mM Tris–HCl pH 7.4, 150 mM NaCl, 5 mM EDTA, 10% glycerol, 1 mM dithiothreitol, and 1 × Halt Protease and Phosphatase Inhibitor Cocktail EDTA-free (Pierce, Thermo Fisher Scientific, Rockford, IL, USA)), followed by sonication and centrifugation at 3200 × *g* during 10 min at 4 °C. Total protein extracts were recovered and stored at −80 °C. Protein concentration was determined by the colorimetric Bradford method (Bio-Rad). BSA (Sigma-Aldrich) was used as standard, and absorbance measurements were performed at 595 nm using the microplate reader (Bio-Rad). To isolate the soluble and detergent-insoluble proteome of BV2 cells, floating and adherent cells were collected in phosphate-buffered saline (PBS)/EDTA, centrifuged at 600 × *g* for 5 min at 4 °C, and the pellet homogenized in NP-40 lysis buffer. Then, cell lysates were rotated for 30 min at 4 °C, and centrifuged at 16,000 × *g* for 20 min at 4 °C. Supernatants were recovered and used as the soluble fractions. To remove carryovers, the pellet was washed with NP-40 lysis buffer and centrifuged again at 16,000 × *g* for 10 min at 4 °C. Urea-sodium dodecyl sulfate (SDS) buffer composed by 8 M urea and 3% SDS in NP-40 lysis buffer was used to resuspend the pellet and followed by sonication. Lysates were then spun at 16,000 × *g* for 20 min at 4 °C, and the supernatants recovered and used as the detergent-insoluble fractions. To determine protein concentration, the bicinchoninic acid (BCA) assay (Thermo Fisher Scientific) was used, according to the manufacturer’s recommendations.

### Immunoblot analysis

Equal amounts of total, insoluble or soluble protein extracts were electrophoretically resolved on 8% SDS-polyacrylamide gels, and transferred onto nitrocellulose membranes. Then, transient staining with 0.2% Ponceau S (Merck, Darmstadt, Germany) was used to confirm protein loading and transfer. Following blocking with 5% milk solution in Tris-buffered saline (TBS), blots were incubated overnight at 4 °C with primary rabbit polyclonal antibodies reactive to RIP1, RIP3, Akt, p-Akt (Ser473), NF-κB p65 and IκBα (#7881, #135170, #8312, #7985, #372, and #371; Santa Cruz Biotechnology, Santa Cruz, CA, USA), MLKL (#M6697; Sigma Aldrich), p-MLKL (Ser358) and p-NF-κB p65 (Ser536) (#196436, #131109; Abcam, Cambridge, UK), p-p38 (Thr180/Tyr182) (#9211; Cell Signaling, Danvers, MA, USA); and with primary mouse monoclonal antibodies reactive to JNK, p-JNK (Thr183/Tyr185),and p38α/β (#7345, #6254, #7972; Santa Cruz Biotechnology) and p-IκBα (Ser32/36) (#9246; Cell Signaling), and finally with secondary goat anti-mouse or anti-rabbit IgG antibody conjugated with horseradish peroxidase (Bio-Rad Laboratories) diluted 1:5000 in blocking solution for 1 h at room temperature. Membranes were processed for protein detection using Immobilon^TM^ Western (Merck Millipore, Burlington, MA, USA) or SuperSignal substrate (Pierce, Thermo Fisher Scientific). β-actin (AC-15; Sigma-Aldrich) was used as endogenous control. Densitometric analysis was performed with the Image Lab Software version 5.1 Beta (Bio-Rad).

### Quantitative RT-PCR

BV2 cells were plated in 12-well plates at 8 × 10^5^ cells/well for real-time RT-PCR analysis. Briefly, total RNA was extracted using TRIzol™ reagent (Invitrogen, Grand Island, USA). RNA was quantified using a Qubit™ 2.0 fluorometer (Invitrogen) and then converted into cDNA using NZY Reverse Transcriptase (NZYTech, Lisbon, Portugal). RT-PCR was performed in an Applied Biosystems 7300 System (Thermo Fisher Scientific). The following primer sequences were used: *COX2* gene, 5′-CAGCCAGGCAGCAAATCCTT (forward) and 5′-AGTCCGGGTACAGTCACACT (reverse); *IL-6* gene, 5′-GACGATACCACTCCCAACAGACC (forward) and 5′-AAGTGCATCATCGTTGTTCATACA (reverse); *NRLP3* gene, 5′-AGAGCCTACAGTTGGGTGAAATG (forward) and 5′-CCACGCCTACCAGGAAATCTC (reverse); and *TNF-α* gene, 5′-AGGCACTCCCCCAAAAGATG (forward) and 5′-TGAGGGTCTGGGCCATAGAA (reverse). Two independent reactions for each primer set were performed in a total volume of 12.5 μl containing 2 × Power SYBR Green PCR master mix (Thermo Fisher Scientific) and 0.3 μM of each primer. The relative amounts of each gene transcript were calculated based on the standard curve normalized to the level of hypoxanthine-guanine phosphoribosyltransferase (*HPRT*) and expressed as fold-change from control cells.

### Enzyme-Linked Immunosorbent Assay (ELISA)

Sandwich ELISA kits (PeproTech) were used to determine TNF-α concentration in culture media. First, the plates were covered with a capture antibody specific for TNF-α overnight at room temperature, followed by removal of the liquid and washing 4 × with washing buffer (0.05% Tween-20 in PBS). Then, blocking buffer (1% BSA in PBS) was added for 1 h at room temperature to block non-specific binding, followed by the same cycle of washes. Afterwards, 100 μL of BV2 cell supernatants were added to each well and incubated for 2 h at room temperature, followed by additional four washes. The detection antibody was then added to each well and incubated for 2 h at room temperature, followed by four washes as before. Avidin peroxidase was finally incubated during 30 min at room temperature, followed by four washes as before. Addition of a peroxidase substrate solution then allows the colourless substrate to convert into a soluble blue coloured product. Colour intensity is proportional to the quantity of TNF-α contained in each sandwich structure. Samples were then incubated at room temperature until green colour was visually detectable (±30 min), followed by absorbance reading at 450 nm, with 590 nm reference wavelengths using a Bio-Rad Model 680 microplate reader. TNF-α concentration (pg/mL) was calculated from standard curves.

### Immunofluorescence

NF-κB p65 nuclear translocation in BV2 cells was examined by immunocytochemistry. Briefly, BV2 cells were incubated with zVAD-fmk in the presence or absence of Nec-1 or Oxa12 for 24 h. Then, cells were fixed with 4% paraformaldehyde in PBS for 20 min at room temperature, followed by two washes with PBS. Nonspecific binding was blocked with 10% normal donkey serum for 1 h at room temperature. Next, cells were incubated with primary mouse monoclonal anti-NF-κB p65 antibody (1:50, Santa Cruz Biotechnology) overnight at 4 °C. Cells were then washed three times with PBS, followed by incubation with Alexa Fluor 568-conjugated donkey anti-rabbit IgG (1:150, Life Technologies) for 2 h at room temperature. Nuclei were stained with Hoechst 33258 (Sigma-Aldrich) and mounted on Mowiol mounting medium (Sigma-Aldrich). Images were taken using a fluorescence microscope.

### Image analysis

BV2 and L929 cells morphology was evaluated by phase-contrast microscopy using a Primo Vert microscope and fluorescence images were captured using an Axio ScopeA.1 fluorescent microscope (Carl Zeiss MicroImaging GmbH, Gottingen, Germany). At least 8 images per condition were acquired using an AxioCam 105 Color camera with the Zen lite 2012 (both from Carl Zeiss MicroImaging GmbH). Quantification of p65 NF-κB signal was performed using ImageJ v3.91 software, by selecting a region of interest according to the localization of the nucleus and measurement of fluorescence intensity in the same region.

### Statistical analysis

All data are presented as mean±standard error the mean (SEM) of at least three independent experiments. Comparison between groups was made by one-way analysis of variance (ANOVA) followed by post hoc Bonferroni’s test. Analysis and graphical presentation were performed with the GraphPad Prism Software version 5.00. The statistical significances were achieved when *p* < 0.05.

## Electronic supplementary material

Suppl Fig 1
