# Supplementary material for: Phenotypic screening identifies a new oxazolone inhibitor of necroptosis and neuroinflammation
Source: Cell Death Discov. 2018 Jul 10;4:65. doi: 10.1038/s41420-018-0067-0 (PMC6060125; doi:10.1038/s41420-018-0067-0)
Supplement: Supplementary file 1 — Suppl Fig 1 [file 41420_2018_67_MOESM1_ESM.docx]

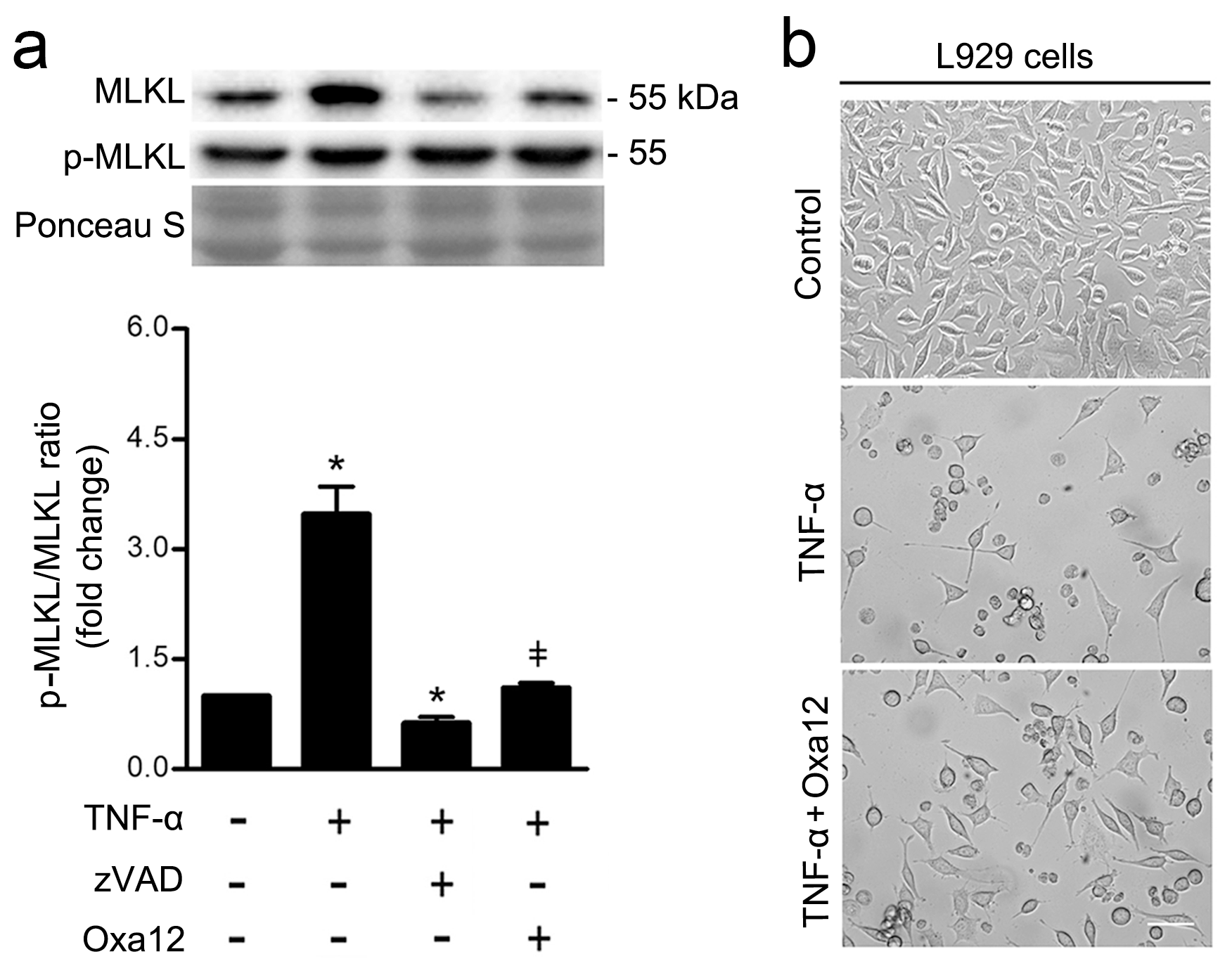


**Supplementary Fig. 1 Oxa12 reduces MLKL phosphorylation in the murine L929 cell line**. **a** L929 cells were incubated with 30 µM each of TNF-α, TNF-α plus Nec-1, or TNF-α plus Oxa12 for 5 h. Total protein lysates were prepared for Western blot analysis of p-MLKL and MLKL. Representative immunoblots are presented with the respective densitometric analysis. Blots were normalized to Ponceau S staining. Values are expressed as mean ± SEM of three independent experiments. **p* ˂ 0.05 vs control; ǂ*p* ˂ 0.05 vs TNF-α. **b** Bright-field microscopic images of L929 cells incubated with 30 µM each of TNF-α, TNF-α plus Nec-1 or TNF-α plus Oxa12 for 24 h. Microscopy images were taken at 100x with a Primo Vert microscope. Scale bar, 100 *µ*m.
